# Comparison of Apolipoprotein (apoB/apoA-I) and Lipoprotein (Total Cholesterol/HDL) Ratio Determinants. Focus on Obesity, Diet and Alcohol Intake

**DOI:** 10.1371/journal.pone.0040878

**Published:** 2012-07-25

**Authors:** Gianluca Tognon, Christina Berg, Kirsten Mehlig, Dag Thelle, Elisabeth Strandhagen, Jaana Gustavsson, Annika Rosengren, Lauren Lissner

**Affiliations:** 1 Public Health Epidemiology Unit, Department of Public Health and Community Medicine, University of Gothenburg, Gothenburg, Sweden; 2 Department of Food and Nutrition, and Sport Science, University of Gothenburg, Gothenburg, Sweden; 3 Section of Biostatistics, Institute of Basic Medical Sciences, University of Oslo, Oslo, Norway; 4 Occupational and Environmental Medicine Unit, Department of Public Health and Community Medicine, University of Gothenburg, Gothenburg, Sweden; 5 Department of Molecular and Clinical Medicine, Institute of Medicine, University of Gothenburg, Gothenburg, Sweden; University of Tor Vergata, Italy

## Abstract

The ratio between apolipoprotein B and apolipoprotein A-I (apoB/apoA-I) has been suggested to be a powerful and more accurate predictor of future cardiovascular disease risk than total cholesterol and HDL cholesterol. Since diet and lifestyle can directly influence dyslipidemia, it is of interest to identify modifiable factors that are associated with high levels of the apolipoprotein ratio and if they can have a different association with a more traditional indicator of cardiovascular risk such as total cholesterol/HDL. The relationship between obesity and dyslipidemia is established and it is of interest to determine which factors can modify this association. This study investigated the cross-sectional association of obesity, diet and lifestyle factors with apoB/apoA-I and total cholesterol/HDL respectively, in a Swedish population of 2,907 subjects (1,537 women) as part of the INTERGENE study. The apolipoprotein and lipoprotein ratios were highly correlated, particularly in women, and obesity was strongly associated with both. Additionally, age, cigarette smoking and alcohol intake were important determinants of these ratios. Alcohol was the only dietary factor that appreciably attenuated the association between obesity and each of the ratios, with a stronger attenuation in women. Other dietary intake and lifestyle-related factors such as smoking status and physical activity had a lower effect on this association. Because the apolipoprotein and lipoprotein ratios share similar diet and lifestyle determinants as well as being highly correlated, we conclude that either of these ratios may be a sufficient indicator of dyslipidemia.

## Introduction

Dyslipidemia, in terms of elevated LDL and triglycerides as well as decreased HDL concentrations, is independently linked to the progression of CVD [1]. However, it has recently been suggested that apolipoproteins (apo) may be more informative risk markers than lipoproteins (e.g. LDL and HDL) [2], particularly, the ratio between apolipoprotein B and apolipoprotein A-I (apoB/apoA-I) [3]–[5] [6]. The measurement error of apolipoproteins is <5% [7], [8] and they are stable in acute stroke [9] while not more expensive to measure than traditional lipoproteins. However the apolipoprotein superiority was not confirmed in all studies [10] and was questioned by some recent important studies such as the Women’s Health Initiative [11], the EPIC-Norfolk [12], [13] and the PREVEND cohort [14]. Obesity (defined as BMI ≥30) is a well-established factor associated with higher levels of apolipoprotein ratio [15], while alcohol intake and regular physical exercise are inversely related to the apolipoprotein ratio [16].

On these premises, the aim of the present study was to identify lifestyle-related correlates of this ratio and potential differences in relation to the more conventional total cholesterol/HDL ratio. Among the investigated lifestyle factors, we focused on obesity, which in contrast to many other cardiovascular risk factors increased sharply in Sweden at the end of the 20^th^ century [17]. In particular, we examined whether any dietary factor could explain the known associations between obesity and either of these ratios and we tested any effect modification by triglycerides on the association between obesity and both ratios. The study population was composed of Swedish adults sampled within the INTERGENE cohort study.

## Methods

### Subjects and Dietary Assessment

INTERGENE is a population based research program that aimed to assess the INTERplay between GENEtic susceptibility and environmental factors for the risk of chronic diseases in western Sweden. The survey started in April 2001 and continued until December 2004. The study population consisted of randomly selected women and men aged 25–74 years living in the Västra Götaland Region at the time of sampling. Of 8,820 individuals invited to participate in the study, 194 were found to be deceased, had moved to another part of Sweden, to another country or had an unknown address. Of the remaining 8,626 eligible individuals, 3,614 (1,915 women) participated yielding a participation rate of 42%. Participants were more likely to be women, of higher age and with a higher education level than non-participants [18]. Due to exclusions described below, the final sample used in this analysis included 2,907 subjects (1,537 women).

Body height and weight were measured to the nearest 1 cm and 0.1 kg, respectively, while the subjects were wearing light clothing and no shoes. Waist circumference was measured at a level midway between the lower rib margin and iliac crest, and hip circumference was measured as the maximum perimeter over the buttocks. 117 subjects had missing values for waist circumference and were thus excluded from the analyses.

Usual dietary intake was assessed with a validated self-administered food frequency questionnaire (FFQ) [19], [20] adapted to the INTERGENE cohort [21] and including 92 frequency questions, 72 of which (including alcoholic drinks) had a choice of 8 intake frequencies ranging from no consumption to three or more occasions per day. For the remaining 20 foods (such as milk and yoghurt with different fat content, beverages other than alcoholic beverages, sugar, cheese and bread of different types), open questions about the number of servings (*e.g.* glasses of milk or juice, cups of tea/coffee, slices of cheese or bread, spoons of sugar) per day or week were included. Missing values for food frequencies were considered equivalent to the indication of no consumption for that specific food item and were thus set to a 0 intake level. Intakes of foods in grams were calculated by multiplying the frequency of consumption of each food item by gender- and age-specific portion sizes [19]. In turn, nutrient and energy intake were estimated by multiplying these values with nutrient content from the Swedish Food Administration Database (1997). Due to implausible energy intake, 155 subjects were excluded from the present analysis. Moreover, 71 subjects were also excluded because they did not fill in the Food Frequency Questionnaire or because the number of missing values for food frequencies was greater than eight.

The Recommended Food Score (RFS) was calculated as described in another Swedish cohort [22] that used the same validated questionnaire later adapted to the INTERGENE cohort (see above [19]–[21]). The RFS is derived from the separation of “healthier” from “less healthy” foods based on their nutrient content, indications from dietary guidelines [23] and results from large epidemiological studies. It includes plant-based food items (fruit, vegetables, nuts), low-fat milk and yoghurt, whole grain bread, crispbreads (high fibre content, no fat) and fish (excluding shellfish). When each recommended food item was consumed 1–2 times per week or more, 1 point was assigned. The final score added up to a maximum of 40 and, in the present analyses, was included on a scale of 10 points.

The questionnaire also included information about health, socioeconomic, and lifestyle factors. Based on these, 7 subjects were excluded because of pregnancy and189 subjects because they were taking statins at enrolment. Physical activity was assessed by means of a validated questionnaire [24] that has been in use in Gothenburg since the 1960s [25]. Each subject was asked to report their physical activity during leisure time, choosing between: 1. Mostly sedentary activities i.e. reading and watching TV, 2. Moderate activities such as walking and cycling (≥4 h/week), 3. Moderate activities (≥5 h/week) or 4. hard training and competitive sports. The subjects were also asked to report their activity level during working time, choosing between: 1. Mostly sedentary, 2. Passive sitting half of time, 3. Mostly standing, 4. Mostly walking/lifting (minor carrying), 5. Mostly walking/lifting (major carrying), 6. Heavy manual labour. PAL values were assessed based on assumed intensity levels of different activities (ratio of work metabolic rate to resting metabolic rate) [26]. Sex-specific PAL quintiles were finally calculated and included in the statistical models.

### Laboratory Analyses

Blood tests were taken at different times of the day on subjects fasting for at least four hours and the blood samples were collected into tubes containing 0.1% EDTA for immediate serum lipid analyses. Serum total cholesterol and triglyceride concentrations were determined using enzymatic assays. HDL cholesterol concentration was measured after dextran sulfate-magnesium precipitation of apoB-containing lipoproteins. Quantitative determination of apoB and apoA-I was done by immunoprecipitation enhanced by polyethylene glycol at 340 nm (Thermo Fisher Scientific, Vantaa, Finland). The analyses were performed on a Konelab 20 auto-analyzer (Thermo Fisher Scientific). Inter-assay coefficient of variation was below 5% for Konelab analyses. 166 subjects were excluded from the analyses because of missing apoB/apoA-I ratio and 2 because of outlier values for this ratio.

### Statistical Analyses

The association between apolipoprotein and lipoprotein ratios was assessed by linear regression analysis separately for men and women as well as in the whole data set including an interaction term with sex. The correlation and 95% Confidence Intervals (95% CIs) between BMI or central adiposity indicators (waist circumference and WHR) [27] and each ratio were assessed in the whole population sample, adjusting for sex. Logistic regression models adjusted for age, sex, smoking status, physical activity, marital status and menopausal status plus estrogen use in women were used to test the statistical interaction between both ratios and triglycerides levels, including obesity as the outcome variable. Higher and lower triglyceride levels were also defined according to sex-specific medians and stratified estimates were produced, of the association between obesity and the ratio(s) whose effect was modified by triglycerides.

**Table 1:** General features of the study population (by sex and overall) including variables describing habits related to alcohol use (abstainers, ethanol intake from different beverages) and outcomes (apolipoprotein and lipoprotein ratios, as well as their components).

	Men (N = 1,370)	Women (N = 1,537)	Whole sample (N = 2,907)
Age (years)	50.6±12.7	50.6±13.1	50.6±12.9
BMI ≥30	209 (15.3%)	213 (13.9%)	422 (14.5%)
Waist circumference ≥88 (♀)/102 (♂) cm	288 (21.0%)	426 (27.7%)	714 (24.6%)
Waist to Hip ratio >0.85 (♀)/1 (♂)	197 (14.4%)	415 (27.0%)	612 (21.0%)
Current cigarette smokers	212 (15.5%)	294 (19.1%)	506 (17.4%)
University education	393 (28.7%)	527 (34.3%)	920 (31.6%)
Physical Activity Level (PAL)	1.7±0.2	1.7±0.2	1.7±0.2
Married or living with partner	1,088 (79.4%)	1,124 (73.1%)	2,212 (76.1%)
Alcohol abstainers	85 (6.2%)	184 (12.0%)	269 (9.2%)
Ethanol intake in consumers (g/day)	12.1±9.7	6.1±4.9	9.1±8.2
Ethanol intake from beer (g/day)	6.2±6.4	1.6±2.2	3.8±5.2
Ethanol intake from wine (g/day)	3.9±4.1	3.7±3.6	3.8±3.9
Ethanol intake from spirits (g/day)	2.1±3.6	0.5±0.8	1.3±2.7
Recommended Food Score (0–40 points)	15.9±5.5	17.9±4.8	17.0±5.3
Total cholesterol/HDL (Lipoprotein ratio)	4.0±1.2	3.2±1.0	3.6±1.2
Total cholesterol (mg/dL)	216±41	214±43	215±42
HDL cholesterol (mg/dL)	57±14	69±17	63±17
ApoB/apoA-I ratio (Apolipoprotein ratio)	0.81±0.23	0.66±0.21	0.73±0.23
Apolipoprotein A-I	1.45±0.22	1.62±0.27	1.54±0.26
Apolipoprotein B	1.14±0.30	1.04±0.29	1.09±0.30
Triglycerides (mg/dL)	138±100	105±56	120±20

For continuous variables, mean value ± standard deviation is given. For prevalences the number of individuals with the property in question is given as well as their percentage within each group.

Multiple linear regression was used to investigate the association between each of the two ratios and age (linearly included on a scale of 10 years), smoking status (current smokers vs non smokers), physical activity (expressed as sex-specific quintiles of the PAL index), marital status (married or living with partner vs all other categories), education (lower education levels vs higher levels) and menopausal status plus estrogen use in women (post-menopausal women not taking estrogens compared with both post-menopausal women taking estrogens and premenopausal women taking estrogens or not), and diet. The latter was investigated both at the food and at the macronutrient level. At the food level we tested the association of both ratios with the Recommended Food Score (included in the model as a continuous variable, on a scale of 10 points). The association of food groups and food items relevant in relation to dyslipidemia was also tested, considering daily intake in grams and adjusting or not for energy intake. At the nutrient level we investigated the association of protein, fat, carbohydrate as well as of different macronutrient subtypes and of ethanol intake, considering daily intake in grams. All variables were included in the models on a continuous scale, divided by 10 in order to get an estimation of the association with an intake increase of 10 g/day. [28]. Ethanol intake was investigated either as a continuous variable (total or beverage-specific intake on a 10 g/day basis) or as a categorical variable based on population-specific tertiles (0.32–4.57 g/day; 4.58–9.78 g/day; >9.78 g/day) including abstainers as the fourth category. Analyses were performed both on the whole dataset (adjusting for sex) and separately for men and women. Natural logarithms of the ratios were used in these models in order to meet the normality assumption of linear regression and reduce skewness.

The parameter estimates from linear regression of log-ratio on predicting variables (β) were presented as percent difference in the outcome for an increase of each covariate by 1 unit (dietary macronutrients were scaled to 10 g/day):
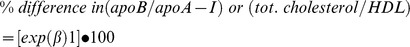



To show the influence of individual covariates (see above) on the association between obesity and each of the two ratios, we calculated how much the regression parameter for obesity adjusted for the covariate (β_obesity|confounder_) differed from the regression parameter for obesity in the unadjusted model (β_obesity_):
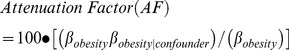



The AF was computed separately for each covariate, and including all confounding variables together in the same model. Positive AF values indicate that the covariate added to the model explains part of the association between obesity and outcome, while negative values indicate that it enhances the association of obesity on outcome, e.g. if the confounder had opposite associations with obesity and outcome.

Finally, sensitivity analyses included adjusting for energy intake (estimated based on macronutrient multiplied by Atwater factors) and the exclusion of subjects reporting previous cardiovascular events or diabetes and subjects abstaining from alcohol consumption where applicable.

### Bioethics

The study was approved by the Ethics Committee of the Medicine Faculty of the University of Gothenburg (Medicinska fakultetens forskningsetikkommitté) in accordance with the Declaration of Helsinki (1989) of the World Medical Association. All participants were informed of the aims and procedures of the study and gave their written consent.

## Results

### Descriptive Results

As reported in Table 1 mean age was 50.6±12.9 years, 14.5% were obese, 17.4% were current cigarette smokers, and 31.6% had a university education. Mean Physical Activity Level (PAL) was 1.7±0.2 in both men and women (range of the lowest sex-specific quintile: 1.30–1.50 in men, 1–35–1.50 in women; range of the highest quintile: 1.90–2.40 in men, 1.90–2.35 in women). Mean ethanol intake among consumers was 9.1±8.2 g/day, with men reporting twice as much ethanol as women (12.1±9.7 g/day vs 6.1±4.9 g/day), while the proportion of abstainers among women was twice that of men. The Recommended Food Score was 2 points lower in men than in women. The latter had higher values of HDL-cholesterol than men and a lower ratio of total cholesterol over HDL-cholesterol (3.2±1.0 in women vs 4.0±1.2 in men). Similarly, the apolipoprotein ratio was higher in men (0.81±0.23) than in women (0.66±0.21).

**Figure 1:**
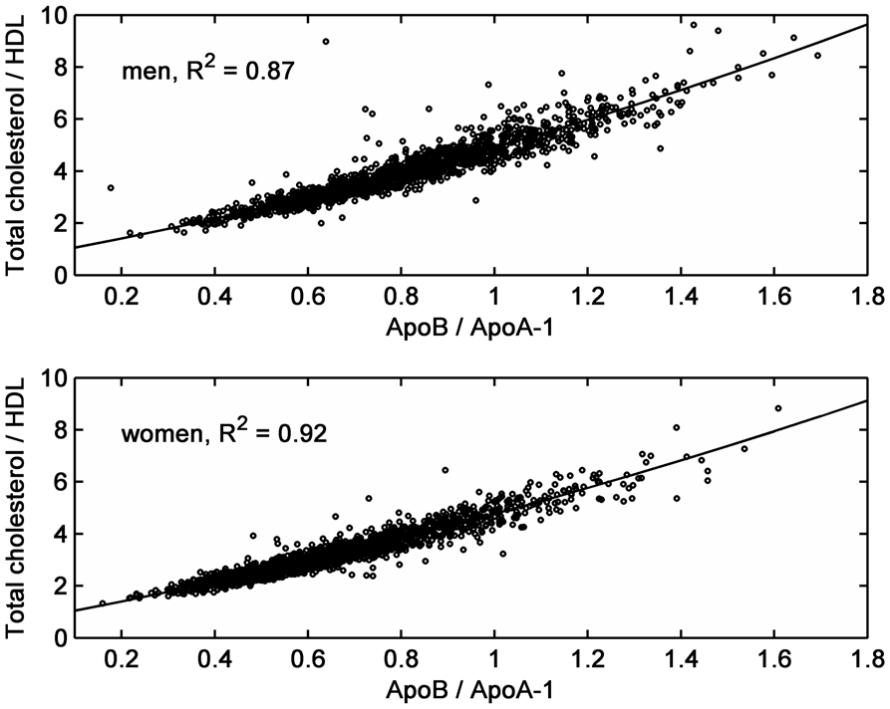
Mutual association between apolipoprotein and lipoprotein ratios. Linear regression, including a quadratic term, of apolipoprotein on lipoprotein ratio, separately for men and women. The regression line and the coefficient of determination (R^2^) are also depicted.

The two ratios were highly correlated (coefficient of determination: R^2^ = 0.87 in men, 0.91 in women, Figure 1) and this correlation was higher in women than in men (p-value for interaction <0.0001), both when excluding or including an outlier in the male cohort characterized by a high lipoprotein ratio coupled to a low apolipoprotein ratio.

### Association of apoB/apoA-I and Total Cholesterol/HDL Ratios with Diet, Lifestyle and Reproductive Factors

The Recommended Food Score was inversely associated with both outcomes, but significantly in women only. Analyses at food level for one food item at a time, adjusted for age, smoking status, physical activity, marital status and menopausal status plus estrogen use in women, showed that two food groups were consistently associated with both ratios in men and women: sweetened products and alcoholic drinks. The former were positively associated with the difference in apolipoprotein ratio (0.4%, 95% CI: 0.1; 0.8 in men and 0.8%, 95% CI: 0.3; 1.4 in women) as well as with the difference in lipoprotein ratio (0.4%, 95% CI: 0.0; 0.8 in men and 0.8%, 95% CI: 0.3; 1.3 in women). On the other hand, alcoholic beverages were inversely associated with the difference in apolipoprotein ratio (−1.1%, 95% CI: −1.7; −0.6 in men and −3.0%, 95% CI: −4.3; −1.7 in women) and in lipoprotein ratio (−1.1%, 95% CI: −1.7; −0.5 in men and −1.9%, 95% CI: −3.1; −0.5 in women). The analyses at macronutrient level summarized the above mentioned results, since sucrose and ethanol intake were associated with both ratios in men and women. Saturated fats showed a slight but significant inverse association with only the apolipoprotein ratio in women.

Although it is not possible to compare all factors on a common scale, Table 2 summarizes the results of a model including different dietary, lifestyle and, in women, reproductive factors, in association with both ratios. For diet we included those variables that in the above mentioned analyses were significantly associated with either ratio, i.e. sucrose and ethanol intake as well as the Recommended Food Score. Saturated fats were also included, considering their *a priori* known relevance as a dietary cardiovascular risk factor. The multivariate results, when RFS, sucrose, saturated fats and alcohol were included in the model at the same time, confirmed what was observed in the above mentioned analyses where each of these factors was singularly tested.

**Table 2:** Gender-stratified association of lifestyle (obesity, low physical activity, smoking status), dietary factors (ethanol, saturated fats, sucrose intake) and other factors (age, education, marital status, menopausal status and estrogen use in women) with both apolipoprotein and lipoprotein ratios (included on a logarithmic scale), expressed as percent (%) change and 95% confidence limits from multiple linear regression models in which all of these covariates were included simultaneously.

	Apolipoprotein ratio (apoB/apoA-I)		Lipoprotein ratio (total cholesterol/HDL)	
	Men (N = 1,370)	Women (N = 1,537)	Men (N = 1,370)	Women (N = 1,537)
	% difference (95% CIs)	% difference (95% CIs)	% difference (95% CIs)	% difference (95% CIs)
BMI (5 units)	14.6 (12.2; 17.0)***	10.8 (9.0; 12.7)***	15.9 (13.3; 18.5)***	11.9 (10.0; 13.7)***
Age (10 yrs††)	3.1 (1.9; 4.4)***	5.3 (3.4; 7.2)***	1.2 (0.0; 2.5)*	3.0 (1.3; 4.7)***
First vs. Second PAL quintile	2.0 (−2.8; 7.1)	−0.6 (−4.4; 3.4)	1.0 (−4.0; 6.2)	−0.5 (−4.3; 3.5)
First vs. Third PAL quintile	4.1 (0.0; 8.4)	0.6 (−3.3; 4.7)	2.4 (−1.9; 6.8)	0.8(−3.1; 4.8)
First vs. Fourth PAL quintile	−0.6 (−4.6; 3.7)	−2.2 (−6.2; 2.1)	−3.1 (−7.3; 1.3)	−1.6 (−5.6; 2.6)
First vs. Fifth (highest) PAL quintile	−6.5 (−10.5; −2.3)**	−0.3 (−4.3; 3.9)	−7.1 (−11.3; −2.7)**	−0.1 (−4.1; 4.0)
Current cigarette smokers	9.7 (5.3; 14.2)***	11.0 (7.2; 14.9)***	8.6 (4.1; 13.4)***	9.6 (6.0; 13.4)***
Low education	−1.2 (−4.9; 2.8)	−0.3 (−4.2; 3.7)	−1.7 (−5.6; 2.3)	0.6 (−3.1; 4.5)
Not married or living with partner	−1.0 (−5.3; 3.5)	−6.4 (−10.4; −2.2)**	−1.4 (−5.9; 3.3)	−6.2 (−10.1; −2.1)**
Ethanol intake (10 g/day††)	−3.5 (−4.9; −2.0)***	−8.9 (−11.4; −6.3)***	−2.9 (−4.4; −1.3)***	−6.3 (−8.8; −3.7)***
Saturated fats (10 g/day††)	−0.5 (−1.4; 0.4)	−1.3 (−2.4; −0.2)**	−0.5 (−1.4; 0.5)	−0.6 (−1.7; 0.4)
Sucrose (10 g/day††)	0.6 (0.2; 1.0)**	0.8 (0.2; 1.4)*	0.5 (0.1; 0.9)*	0.9 (0.3; 1.5)**
Recommended Food Score†	0.7 (−2.0; 3.4)	−2.7 (−5.5; 0.1)	−0.5 (−3.3; 2.4)	−3.5 (−6.2; −0.7)*
Post-Menopause (no)/Estrogen use (no) †††	–	−2.8 (−7.5; 2.2)	–	−2.7 (−7.5; 2.0)
Post-Menopause (no)/Estrogen use (yes) †††	–	−6.8 (−11.6; −1.7)**	–	−5.5 (−10.2; −0.6)*
Post-Menopause (yes)/Estrogen use (yes) †††	–	−2.9 (−6.5; 0.8)	–	−2.0 (−5.5; 1.7)

* p<0.05,

** p<0.01,

*** p<0.001;

† Expressed as an increase in 10 points.

†† These variables were included continuously, divided by 10 in order to get an estimation of the effect on a scale of 10 units (e.g.: 10 years or 10 g/day).

††† Reference category: Post-Menopause (yes)/Estrogen use (no).

Other significant positive associations were found for cigarette smoking, for the highest (vs. the lowest) sex-specific quintile of physical activity in men and for living with a partner (vs not) in women. In women, lower ratio levels were observed in premenopausal women taking estrogens compared to post-menopausal women who did not take estrogens (the reference category). Notably, BMI showed a very strong positive association with both ratios.

### Role of Different Factors in Explaining the Association between Obesity and Both Ratios

Table 3 shows how different factors related to cardiovascular risk could individually attenuate the association observed between obesity and both ratios. In men, the highest attenuation factors were observed for age and ethanol intake (both ratios) as well for diet (lipoprotein ratio only), while in women the highest attenuation factors were observed for age, reproductive variables and ethanol intake, for both ratios. The overall attenuation exercised by all factors together was equal to 13.2% (apolipoprotein ratio) and 11.2% (lipoprotein ratio) in men and 28.5% (apolipoprotein ratio) and 21.5% (lipoprotein ratio) in women.

**Table 3:** Role of different factors associated with higher levels of apoB/apoA-I potentially able to explain the association, in both men and women, between obesity (defined as BMI ≥30 kg/m^2^) and both apolipoprotein (apoB/apoA-I) and lipoprotein (total cholesterol/HDL) ratios (included on a logarithmic scale) and expressed as percent (%) change and 95% confidence limits.

Independent variables	% difference in apoB/apoA-I(95% CIs)	AF	% difference in total cholesterol/HDL(95% CIs)	AF
**Men (N = 1,370)**				
Obesity (unadjusted)	20.3 (15.3; 25.5)	–	21.8 (16.5; 27.4)	–
Model 1: obesity plus age	18.9 (14.0; 24.0)	6.3	21.0 (15.8; 26.6)	3.2
Model 2: obesity plus ethanol	19.6 (14.7; 24.8)	2.9	21.2 (16.0; 26.7)	2.5
Model 3: obesity plus smoking status	20.0 (14.9; 25.0)	1.6	21.5 (16.2; 27.0)	1.4
Model 4: obesity plus diet*	20.0 (15.0; 25.1)	1.5	21.3 (16.0; 26.8)	2.1
Model 5: obesity plus education	19.9 (15.0; 25.1)	1.5	21.7 (16.4; 27.2)	0.6
Model 6: obesity plus physical activity	21.0 (15.5; 26.8)	0.4	22.8 (17.2; 28.5)	0.9
Model 7: obesity plus marital status	20.1 (15.2; 25.3)	0.6	21.7 (16.4; 27.2)	0.5
Fully adjusted model†	17.9 (12.6; 23.3)	13.2	20.5 (15.2; 26.1)	11.2
**Women (N = 1,537)**				
Obesity (unadjusted)	24.0 (18.6; 29.6)	–	24.3 (19.2; 29.7)	–
Model 1: obesity plus age	18.9 (14.0; 23.9)	19.6	20.3 (15.4; 25.3)	15.2
Model 2: obesity plus reproductive variables	20.8 (15.9; 26.0)	11.9	21.7 (16.9; 26.8)	9.7
Model 3: obesity plus ethanol	21.1 (16.0; 26.5)	10.8	22.3 (17.3; 27.5)	7.7
Model 4: obesity plus education	22.8 (17.5; 28.3)	4.5	23.3 (18.2; 28.6)	3.8
Model 5: obesity plus diet*	23.0 (18.0; 28.8)	2.6	23.7 (18.6; 29.0)	2.3
Model 6: obesity plus physical activity	26.8 (20.6; 33.3)	−1.6	25.4 (19.9; 31.2)	1.0
Model 7: obesity plus marital status	24.1 (18.8; 29.7)	−0.6	24.5 (19.4; 29.8)	−0.5
Model 8: obesity plus smoking status	24.4 (19.0; 29.9)	−1.4	23.3 (18.2; 28.6)	−1.6
Fully adjusted model†	19.9 (14.4; 25.6)	28.5	20.2 (15.2; 25.4)	21.5

The Attenuation Factor (AF) expresses the percentage reduction of the association between obesity (BMI ≥30) and apoB/apoA-I after the association between obesity and the outcome is singularly adjusted for each covariate or for all the covariates at the same time (fully adjusted model).

* Includes saturated fat and sucrose intake as well as the Recommended Food Score.

† Adjusted for age, physical activity, smoking status, education, marital status, ethanol, diet (saturated fats, sucrose, Recommended Food Score) and reproductive variables in women (menopausal status and hormone use).

In logistic regression models testing the association between both ratios and obesity, a statistically significant interaction between triglycerides and the lipoprotein ratio was found in men and women combined, showing a stronger association of the lipoprotein ratio and obesity at lower triglyceride levels defined according the sex-specific median (OR = 1.67, 95% CI: 1.26; 2.21 at lower triglyceride levels; OR = 1.45, 95% CI: 1.29; 1.63 at higher levels).

### Association with Ethanol and Alcoholic Beverages

**Figure 2:**
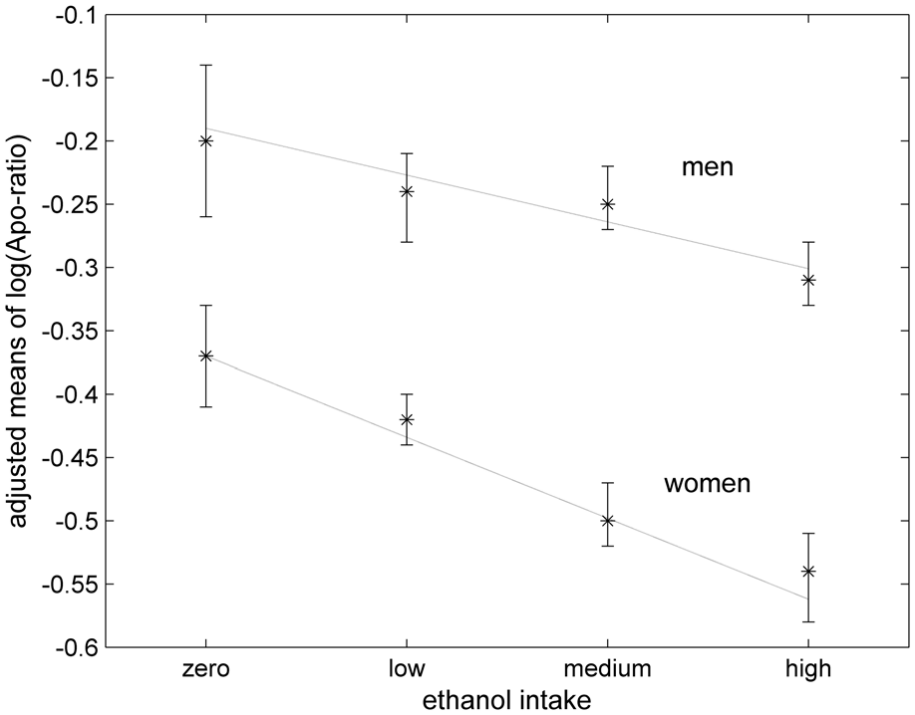
Relationships between the apolipoprotein ratio and ethanol intake, separately for men and women. Mean values for log(apolipoprotein ratio) by tertiles of ethanol intake (0.32–4.57 g/day; 4.58–9.78 g/day; >9.78 g/day) and abstainers, including an interaction with sex and adjusted for age and BMI. The lines were obtained from linear regression of the dependent variable on increasing categories of ethanol intake and only meant to guide the eye.

Total ethanol intake was inversely related with both outcomes in all analyses, with a stronger association in women (p-value for interaction <0.001 for both ratios) (Table 4). The inverse association between both ratios and ethanol intake was mainly explained by consumption of wine in women and of beer and wine in men (Tables 1, 2, 3, 4). In spite of lower prevalence and lower absolute consumption, ethanol from spirits was found to reduce the apolipoprotein ratio in women but not in men. A decreasing trend of the mean values of (log)apolipoprotein ratio with increasing ethanol intake categories (adjusted for age and BMI) was more pronounced in women than in men (p-value for interaction  = 0.04) (Figure 2). No gender difference in the effect of BMI on apolipoprotein ratio were found when ethanol was taken into account.

**Table 4:** Association of total alcohol intake (alone, in a first model) and ethanol from difference sources (simultaneously included in a second model) with percent (%) difference in both the apolipoprotein (apoB/apoA-I) and the lipoprotein (total cholesterol/HDL) ratio (included on a logarithmic scale), from a multiple linear regression model adjusted for age, obesity, physical activity, smoking status, education, marital status, saturated fat intake, sucrose intake, the Recommended Food Score, gender (in combined analyses) and menopausal status plus estrogen use (in women).

	Alcohol users (N)	Men		Users (N)	Women		Alcohol users (N)	Whole sample	
Apolipoprotein ratio		%	95% CIs		%	95% CIs		%	95% CIs
Alcohol (10 g/day)	1,284	−3.7	(−5.2; −2.2) ***	1,348	−9.8	(−12.3; −7.1) ***	2,632	−5.2	(−6.5; −3.9) ***
Alcohol from wine (10 g/day)	1,050	−4.7	(−8.3; −1.0) *	1,242	−12.7	(−16.2; −9.0) ***	2,292	−9.1	(−11.6; −6.5) ***
Alcohol from beer (10 g/day)	1,213	−3.5	(−5.9; −1.1) **	965	−0.1	(−6.7; 7.0)	2,178	−3.0	(−5.2; −0.6) *
Alcohol from spirits (10 g/day)	950	−2.7	(−7.0; 1.8)	568	−19.3	(−33.0; −2.9) *	1,518	−4.1	(−8.2; 0.2)
**Lipoprotein ratio**									
Alcohol (10 g/day)	1,284	−3.1	(−4.6; −1.4) ***	1,348	−7.6	(−10.2; −4.9) ***	2,632	−4.1	(−5.4; −2.8) ***
Alcohol from wine (10 g/day)	1,050	−3.7	(−7.6; 0.4)	1,242	−10.6	(−14.2; −6.8) ***	2,292	−7.3	(−10.0; −4.6) ***
Alcohol from beer (10 g/day)	1,213	−4.0	(−6.6; −1.4) **	965	2.2	(−4.4; 9.2)	2,178	−3.1	(−5.4; −0.7) *
Alcohol from spirits (10 g/day)	950	0.5	(−4.9; 6.3)	568	−15.3	(−29.4; 1.6)	1,518	−1.1	(−6.0; 4.1)

All ethanol and alcohol beverage variables were included as continuous variables in the models, divided by 10 in order to get an estimation of the effect on a scale of 10 g/day.

* p<0.05,

** p<0.01,

*** p<0.001.

Moreover, total ethanol intake was positively associated with apoA-I in both men and women and inversely associated with apoB in women. In contrast, no association was found for total cholesterol while HDL was positively associated with ethanol intake (see Table S1).

No interaction between ethanol intake and smoking status was found and the adjusted means of both ratios were consistent between ever and never smokers across different ethanol intake levels (Table S2).

### Sensitivity Analyses

The main analyses were repeated and the results confirmed after excluding subjects reporting previous cardiovascular events or diabetes (N = 70). Results on ethanol and alcoholic drinks were unaffected after excluding abstainers (N = 269) or after adjusting for energy intake (kcal/day).

## Discussion

In this analysis we sought to identify possible differences in dietary and lifestyle factors associated with the apoB/apoA-I ratio in comparison to the total cholesterol/HDL ratio. The apolipoprotein and lipoprotein ratios were highly correlated (R^2^ = 0.9) and indeed, they showed similar correlates. In other studies, the correlation with the lipoprotein ratio was not as high as in this study [2] or the apolipoprotein ratio was compared with that of non-HDL cholesterol/HDL and not with the more appropriate total cholesterol/HDL ratio [4].

BMI was a strong modifiable risk factor for high values of both ratios but notably, with the exception of alcohol, dietary intake played no, or only a minor influence on the association between obesity and both ratios and was in itself only marginally related to them. Additionally, none of the potential confounders we studied could explain the association between obesity and the two ratios, in line with the well-known fact that obesity is an independent risk factor for atherosclerosis [29]. The only difference we found was an effect modification by triglycerides on the association between obesity and the lipoprotein but not the apolipoprotein ratio, with a stronger association at lower triglyceride values. Our data suggest that in obese subjects, most dietary and lifestyle features reported in questionnaires are uninformative determinants as such of increased levels of either the apolipoprotein or the lipoprotein ratio. Alternatively, the correlation between obesity and dietary habits could have made it impossible to capture any influence of diet on either the studied outcomes. However, the difference in diet quality between obese and non-obese was very low (<1 on a scale of 40 in the RFS), indicating that the above mentioned result was not explained by the difference in diet quality captured by the RFS. The same can be stated for physical activity, since no significant difference in activity levels emerged comparing the obese with the rest of the population. On the other hand, we acknowledge that it is equally possible that other measuring tools which are more accurate and more resistant to reporting bias in obese compared to food frequency questionnaires are needed. Ethanol was found to be an important exception, being inversely and strongly associated with both ratios, as well as being able to reduce the intensity of the association between obesity and both ratios, although it was not assessed with more detail than other single diet components assessed by the FFQ. Alcoholic drinks are expected to be more frequently underreported than overreported as the intakes get higher and this would be more likely to produce a weaker association, while in our study the association between ethanol and both ratios was strong and significant. On the other hand, obesity can also be considered a potential confounding factor related to both exposure (ethanol) as well as to dyslipidemia. Obese subjects had lower alcohol intake compared to non-obese subjects and due to the increased adiposity, they are expected to have a lower water-to-fat ratio than leaner subjects and thus need to drink less to achieve a given ethanol concentration sufficient to influence blood lipid levels. In our study sample alcohol intake was related to both the outcome (apo) and the exposure (BMI >30). The association of ethanol with both ratios was not influenced by total energy intake and, relevantly, the association was more pronounced in women than in men. For apolipoprotein, this result could be due to the observation that in women ethanol intake influenced both apolipoproteins included in the ratio, while in men the intake was associated with apolipoprotein B only. Women are known to metabolize ethanol less efficiently than men and are thus generally considered at increased risk of alcohol related damage than men [30]. Moreover, different beverages seemed here to have differential associations, although the influence of alcoholic beverages on dyslipidemia and CVD risk have generally been found to be independent of beverage type [31], [32]. Therefore, this remains an open question [33], also considering that differential effects related to the beverage type have been observed for other medical conditions [34]. In particular, the intake of alcohol was found to be inversely associated with apoB/apoA-I in a study sub-sample of post-menopausal 64-years old women, two thirds of whom were diabetic or had impaired glucose tolerance [16].

The present study has both strengths and limitations. Among the former we list the fact that it is a population-based cohort including both men and women where weight status, cardiovascular risk factors and dietary intakes have been assessed as previously documented in detail [35]–[38]. Other strengths include the availability of data concerning various potential confounders, the possibility to capture different aspects of diet and particularly to discriminate ethanol intake from different beverages, the availability of both apolipoprotein and lipoprotein measures from samples collected on the same occasion and finally a sufficient study population size to allow analyses on men and women separately. Among the limitations we acknowledge the cross-sectional design that does not allow inference on possible causative relationships but only on associations. This could be associated to a reverse causation bias, particularly in the relationships between obesity, ethanol intake and dyslipidemia. Furthermore, the diet data was based on food frequency questionnaires which were semi-quantitative with regard to quantities consumed and thus likely to contain obesity-related underreporting. Information bias in questionnaires on diet and lifestyle is inevitable and can have partially influenced the calculation of attenuation of diet and lifestyle variables in modulating the association between obesity and dyslipidemia. In particular, an underestimation of the effect of diet and lifestyle on dyslipidemia due to socio-demographic differences between participants and non-participants is also possible [18]. Finally, both smoking status and obesity were both strongly associated with dyslipidemia so we cannot exclude some residual confounding by these factors.

In conclusion, our study showed that apolipoproteins and lipoproteins shared common determinants. A positive association of alcoholic beverages on the lipid profile has also been confirmed for both ratios and was stronger in women than in men. With the exception of ethanol, diet played no, or only a minor part, in explaining the association of obesity with either ratio. A follow up study on INTERGENE is planned and will give the opportunity to study more in detail the differences between these two ratios and their interaction with triglyceride levels on CVD risk.

## Supporting Information

Table S1 Association of total ethanol intake (included as a continuous variable in the models, on a scale of 10 g/day as in previous analyses) with the components of both the apolipoprotein and lipoprotein ratios (on a logarithmic scale). The analyses were performed by means of multiple linear regression models adjusted for age, obesity, physical activity, smoking status, education, marital status, saturated fat intake, sucrose intake, the Recommended Food Score and menopausal status plus estrogen use (in women). Percent (%) change and 95% Confidence Intervals of each (apo)lipoprotein are shown.
(DOCX)

Table S2 Adjusted means of both the apolipoprotein and lipoprotein ratios (on a logarithmic scale) across categories of ethanol intake and stratified by smoking status. Adjusted mean values were obtained from multiple linear regression models adjusted for gender, age, BMI, physical activity, smoking status, education, marital status, saturated fat intake, sucrose intake and the Recommended Food Score.
(DOCX)
