# Supplementary material for: Comparison of Apolipoprotein (apoB/apoA-I) and Lipoprotein (Total Cholesterol/HDL) Ratio Determinants. Focus on Obesity, Diet and Alcohol Intake
Source: PLoS One. 2012 Jul 25;7(7):e40878. doi: 10.1371/journal.pone.0040878 (PMC3405058; doi:10.1371/journal.pone.0040878)
Supplement: Table S1 — Association of total ethanol intake (included as a continuous variable in the models, on a scale of 10 g/day as in previous analyses) with the components of both the apolipoprotein and lipoprotein ratios (on a logarithmic scale). The analyses were performed by means of multiple linear regression models adjusted for age, obesity, physical activity, smoking status, education, marital status, saturated fat intake, sucrose intake, the Recommended Food Score and menopausal status plus estrogen use (in women). Percent (%) change and 95% Confidence Intervals of each (apo)lipoprotein are shown. (DOCX) [file pone.0040878.s001.docx]

**Table S1:** Association of total ethanol intake (included as a continuous variable in the models, on a scale of 10 g/day as in previous analyses) with the components of both the apolipoprotein and lipoprotein ratios (on a logarithmic scale). The analyses were performed by means of multiple linear regression models adjusted for age, obesity, physical activity, smoking status, education, marital status, saturated fat intake, sucrose intake, the Recommended Food Score and menopausal status plus estrogen use (in women). Percent (%) change and 95% Confidence Intervals of each (apo)lipoprotein are shown.

|  | **% difference (95% CIs)** | | | |
| --- | --- | --- | --- | --- |
|  | **Apolipoprotein B** | **Apolipoprotein A-I** | **Total cholesterol** | **HDL-Cholesterol** |
| Ethanol (10 g/day, men) | -0.2 (-1.6; 1.1) | 3.5 (2.7; 4.3) | 0.9 (-0.2; 2.0) | 4.1 (2.7; 5.5) |
| Ethanol (10 g/day, women) | -4.6 (-7.0; -2.3) | 6.0 (4.3; 7.7) | 0.8 (-1.2; 2.7) | 9.4 (6.7; 12.2) |
