# Supplementary material for: Comparison of Apolipoprotein (apoB/apoA-I) and Lipoprotein (Total Cholesterol/HDL) Ratio Determinants. Focus on Obesity, Diet and Alcohol Intake
Source: PLoS One. 2012 Jul 25;7(7):e40878. doi: 10.1371/journal.pone.0040878 (PMC3405058; doi:10.1371/journal.pone.0040878)
Supplement: Table S2 — Adjusted means of both the apolipoprotein and lipoprotein ratios (on a logarithmic scale) across categories of ethanol intake and stratified by smoking status. Adjusted mean values were obtained from multiple linear regression models adjusted for gender, age, BMI, physical activity, smoking status, education, marital status, saturated fat intake, sucrose intake and the Recommended Food Score. (DOCX) [file pone.0040878.s002.docx]

**Table S2:** Adjusted means of both the apolipoprotein and lipoprotein ratios (on a logarithmic scale) across categories of ethanol intake and stratified by smoking status. Adjusted mean values were obtained from multiple linear regression models adjusted for gender, age, BMI, physical activity, smoking status, education, marital status, saturated fat intake, sucrose intake and the Recommended Food Score.

| **Ethanol intake** | **Log(ApoB/apoA-I)** | | **Log(Total cholesterol/HDL)** | |
| --- | --- | --- | --- | --- |
|  | **Never smokers** | **Ever smokers** | **Never smokers** | **Ever smokers** |
| Abstainers | -0.32 (-0.37; -0.27) | -0.23 (-0.29; -0.17) | 1.27 (1.22; 1.32) | 1.37 (1.31; 1.42) |
| Low intake | -0.35 (-0.38; -0.32) | -0.32 (-0.35; -0.29) | 1.25 (1.22; 1.27) | 1.27 (1.24; 1.30) |
| Medium intake | -0.38 (-0.41; -0.35) | -0.38 (-0.41; -0.35) | 1.22 (1.19; 1.25) | 1.24 (1.21; 1.27) |
| High intake | -0.44 (-0.47; -0.41) | -0.40 (-0.43; -0.37) | 1.18 (1.15; 1.21) | 1.20 (1.18; 1.23) |
